# Rates of Adjacent Segment Disease in Polyetheretherketone Versus Titanium Rods After Posterior Lumbar Fusion: A Systematic Review and Meta-Analysis

**DOI:** 10.7759/cureus.85080

**Published:** 2025-05-30

**Authors:** Nicolas K Goff, Landon S Ashby, Justin N Jensen, Logan Muzyka, Michael T Koltz

**Affiliations:** 1 Department of Neurological Surgery, University of Texas at Austin Dell Medical School, Austin, USA; 2 Department of Orthopedic Surgery, University of Texas at Austin Dell Medical School, Austin, USA

**Keywords:** adjacent segment disease (asd), polyetheretherketone (peek), posterior lumbar fusion, systematic review and meta-analysis, titanium rods

## Abstract

Adjacent segment disease (ASD) is a common complication and cause of reoperation following lumbar spinal fusion. Although most commonly performed with titanium or other metal rods, the use of polyetheretherketone (PEEK) rods in spinal fusion has increased. It has been hypothesized that the material properties of PEEK, compared to titanium, allow for less strain on, and therefore less degeneration of, segments adjacent to the fused segments. A systematic search of PubMed and ScienceDirect returned 967 abstracts, of which 13 full-text articles were included in the PEEK rod group and 12 articles were included in the titanium rod group. The two cohorts had similar sex distributions, although the PEEK rod group was significantly younger (61.1 years vs. 62.5 years, p < 0.0001). A meta-analysis using a random effects model with restricted maximum likelihood estimation resulted in an overall incidence rate of 0.026 cases per person-year in the PEEK rod group (95% confidence interval (CI) = 0.010-0.043) and an overall incidence rate of 0.024 cases per person-year in the titanium rod group (95% CI = 0.014-0.033). One-way analysis of variance revealed no significant difference in the incidence rates of ASD between the two groups (p = 0.8422). In conclusion, there is no evidence of any difference in the rate of ASD when performing lumbar fusions with PEEK rods versus titanium rods.

## Introduction and background

Adjacent segment disease (ASD) is a common complication of spinal fusion surgery involving degeneration of the spinal cord segment directly above or below a fused segment [1]. The clinical presentation of ASD can vary widely and includes symptoms such as segmental instability, vertebral compression fractures, disc herniation, facet arthropathy, or listhesis. It is believed to arise from altered biomechanical stress distribution resulting from rigid fixation, which places increased strain on adjacent unfused motion segments [2].

ASD can be classified into two major types, namely, clinical ASD and radiographic ASD. Clinical ASD refers to symptomatic deterioration manifesting as pain or neurological deficits, while radiographic ASD describes degenerative changes detected through imaging, which may or may not be accompanied by clinical symptoms [3]. This distinction is critical for the interpretation of incidence and outcomes across studies.

The incidence of ASD after lumbar spinal fusion remains highly variable in the literature. A 2014 systematic review estimated a range between 2% and 14% depending on diagnostic criteria, follow-up duration, and surgical technique [4]. Patient-specific factors such as older age, female sex, osteoporosis, postmenopausal status, prior facet joint injury, and preexisting disc degeneration further influence ASD risk [3]. Additionally, surgical factors such as the number of levels fused, sagittal alignment correction, and the type of instrumentation play significant roles.

Among these surgical factors, the material properties of the fusion hardware are an emerging focus of investigation. A prior meta-analysis by Seaman et al. compared titanium and polyetheretherketone (PEEK) interbody fusion implants and found no clear superiority, underscoring the ongoing debate over optimal implant material [5]. In contrast, semi-rigid systems using PEEK, AccuFlex, or Isobar rods have been proposed to reduce biomechanical stress at adjacent levels. A computational model comparing rigid and semi-rigid systems indicated that semi-rigid rods may better preserve adjacent facet joint integrity, particularly at the upper levels [6].

PEEK implants offer several attractive features, including a modulus of elasticity more closely matched to cortical bone, radiolucency on imaging, and biocompatibility. However, they may be prone to micromotion and do not osseointegrate as readily as metallic implants. Titanium, by contrast, offers superior strength and stability but can introduce imaging artifacts and may contribute to stress shielding.

Despite growing interest in alternative materials, there is limited clinical evidence comparing ASD rates between patients receiving PEEK versus titanium rods. The present study aims to address this gap by evaluating whether rod material influences the development of ASD following lumbar spinal fusion. Specifically, this meta-analysis compares the incidence of ASD between patients treated with PEEK rods and those treated with traditional titanium rods.

## Review

Methodology

Search Strategy and Study Selection

This meta-analysis followed the Preferred Reporting Items for Systematic Reviews and Meta-Analyses (PRISMA) guidelines [7] and was registered on PROSPERO (CRD42023423034). Electronic searches were conducted in PubMed and ScienceDirect using Boolean combinations of the following terms: “PEEK,” “titanium,” “adjacent segment disease,” “lumbar fusion,” and “spinal instrumentation.” Searches were limited to studies published in English through February 2024.

The publication range for studies included in this meta-analysis spanned from 2010 to 2023, with most PEEK-related studies published between 2010 and 2020 and titanium studies extending through 2023. This range was chosen to include the breadth of available clinical evidence while capturing recent advancements in implant technology.

Inclusion criteria included (1) prospective or retrospective clinical studies, (2) clearly specified rod material (PEEK or titanium), (3) reported incidence of ASD, (4) lumbar spine surgeries, and (5) minimum one-year follow-up. Exclusion criteria included finite element studies, reviews, commentaries, and non-clinical literature.

Data Extraction and Definitions

All studies identified by our search terms were loaded into Rayyan for abstract and full-text review. All abstract and subsequent full-text screening was performed independently by NKG, JJ, and LA. All conflicts were resolved by discussion between the three screening authors. Data extraction was performed by NKG and checked by JJ and LA.

The following data were extracted from studies included in the meta-analysis: title, author, year of publication, country of origin, sample size, mean or median age of participants, percentage of males in the study population, average follow-up time, the number of ASD cases, the number of revision surgeries, and the number of overall complications.

Titles and abstracts were reviewed in Rayyan by NKG, JJ, and LA independently. Discrepancies were resolved via discussion between the three screening authors. Data extraction was performed by NKG and checked by JJ and LA. Extracted variables included study title, authorship, country, publication year, patient demographics, follow-up duration, number of ASD cases, revision surgeries, and reported complications.

Statistical Analysis

Two-tailed z-tests were performed to determine if there were significant differences in mean age, follow-up duration, or the proportion of male patients between the PEEK and titanium cohorts. In instances where studies did not report standard deviations for age, estimates were derived using the method described by Walter and Yao [8]. For each study, the incidence rate of ASD was calculated in cases per person-year, and the standard error was estimated using the rule of three [9]. Incidence rates for each group were meta-analyzed using JASP software, employing a random effects model with restricted maximum likelihood estimation [10]. The resulting pooled estimates were reported with 95% confidence intervals (CIs) and visualized using Forest plots. Between-study heterogeneity was assessed using the I² statistic. Publication bias was evaluated through funnel plots and the Rank correlation test for asymmetry. Finally, a one-way analysis of variance (ANOVA) was conducted to determine if the difference in ASD incidence rates between the PEEK and titanium groups was statistically significant at the α = 0.05 level.

Results

**Figure 1:**
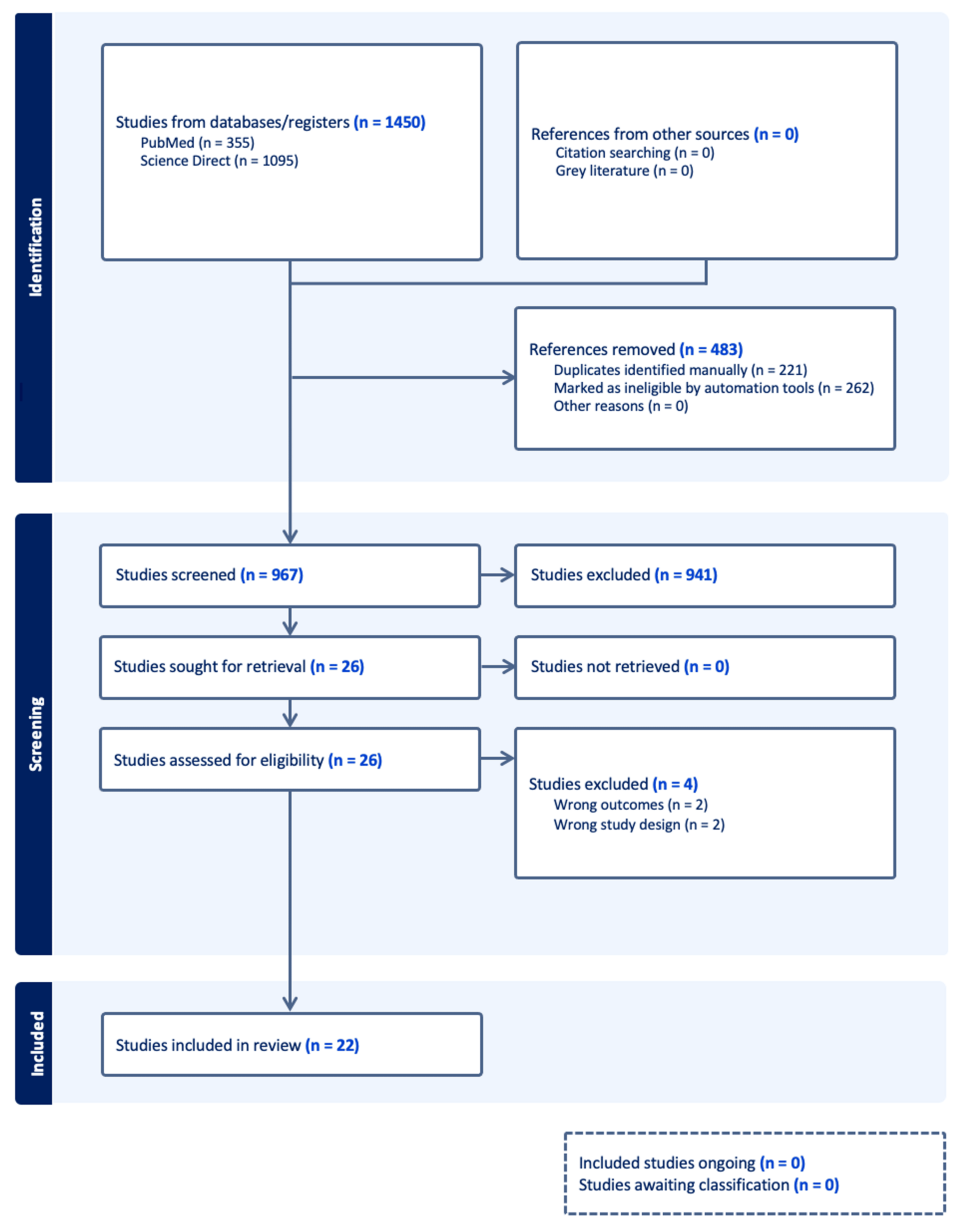
Preferred Reporting Items for Systematic Reviews and Meta-Analyses (PRISMA) flowchart used in the literature review.

The search of PubMed and ScienceDirect yielded 1,450 records, which resulted in 967 unique records after removing duplicates. Of these, the authors selected 26 abstracts for full-text review, 14 related to the PEEK rod group and 12 related to the titanium rod group. After full-text evaluation, four studies (two from each group) were excluded. Ultimately, 22 studies were included in the meta-analysis: 10 in the PEEK rod group, 9 in the titanium rod group, and 3 studies that applied to both groups. The complete review process is illustrated in Figure 1.

The PEEK rod group consisted of 13 studies, including 972 patients, with a mean age of 61.1 years. This group was 44.5% male, and the mean follow-up time was 3.91 years. There were 97 cases of ASD in this group, for a cumulative incidence of 9.98%. The titanium rod group consisted of 12 studies, including 3,622 patients. The mean age of this group was 62.5 years, the mean follow-up time was 4.23 years, and the group was 43.5% male. In this group, there were 356 ASD cases, and the cumulative incidence was 9.83%. There was no significant difference in the proportion of males between the two groups (Z = 0.539, p = 0.5892). There was, however, a significant difference in the mean ages of the two cohorts (Z = -4.002, p < 0.0001), as well as between the mean follow-up times (Z = 12.733, p < 0.001). For three studies, two in the PEEK rod group and one in the titanium rod group, there was neither a standard deviation nor a range reported for age, in which case the standard deviation was assumed to be the mean standard deviation for the rest of the group. Descriptive characteristics of the individual studies included in both groups are presented in Table 1.

**Table 1:** Descriptive characteristics for each study and overall group characteristics. ASD: adjacent segment disease; m: months; n: number of patients; PEEK: polyetheretherketone; Ti: titanium; y: years

	Author	Year	Country	Evidence level	n	Male %	Average age	Average follow-up	ASD cases	ASD rate
PEEK rod	Qi et al. [11]	2013	China	II	20	81.80%	50.4	1 y	0	0
	Athanasakopoulos et al. [12]	2013	Greece	IV	52	44.20%	55.4	3 y	0	0
	Colangeli et al. [13]	2015	Italy	IV	12	75.00%	43.25	29.1 m	0	0
	Ormond et al. [14]	2016	USA	IV	42	59.50%	53.7	31.4 m	5	0.0455
	Oikomonidis et al. [15]	2018	Germany	II	22	27.30%	57.6	2 y	3	0.0682
	Ogrenci et al. [16]	2019	Turkey	IV	172	27.90%	55.6	62.7 m	4	0.0045
	Tosic et al. [17]	2019	Switzerland	IV	20	25.00%	72.5	1 y	1	0.05
	Krieg et al. [18]	2019	Germany	IV	322	49.10%	69.1	51.6 m	60	0.0433
	Dalbayrak et al. [19]	2020	Turkey	IV	48	75.00%	67.08	48.3 m	0	0
	Hirt et al. [20]	2021	USA	IV	154	44.20%	59.9	5 y	13	0.0169
	Koban et al. [21]	2021	Turkey	IV	16	25.00%	58.63	38.8 m	2	0.0387
	Zhao et al. [22]	2022	China	IV	28	64.30%	44.8	2 y	0	0
	Varol et al. [23]	2022	Turkey	IV	64	25.00%	55.25	1 y	9	0.1406
	Total	-	-	-	972	44.49%	61.1	3.91	97	-
Ti rod	Qi et al. [11]	2013	China	II	21	52.40%	48.9	1 y	0	0
	Lee et al. [24]	2015	South Korea	IV	256	80.90%	54.7	1 y		0
	Herren et al. [25]	2018	Germany	II	14	42.90%	61.8	37.68 m	4	0.091
	Hayashi et al. [26]	2018	Japan	IV	20	25.00%	69.3	2 y	1	0.025
	Herren et al. [27]	2018	Germany	II	14	28.60%	65.5	2 y	3	0.1071
	Prassas et al. [28]	2021	Greece	III	77	42.90%	62.6	1 y	4	0.0519
	Samal et al. [29]	2021	Czech Republic	IV	64	64.10%	58.9	2 y	2	0.0156
	Alvarez et al. [30]	2021	USA	IV	153	39.90%	66.6	1 y	0	0
	Koban et al. [21]	2021	Turkey	IV	42	26.20%	59	40.4 m	8	0.0566
	Hirt et al. [20]	2021	USA	IV	2844	40.70%	63.2	5 y	315	0.0222
	Sekiguchi et al. [31]	2022	Japan	IV	34	50.00%	67	46 m	0	0
	Varol et al. [23]	2022	Turkey	IV	63	23.50%	57.7	1 y	19	0.3016
	Total	-	-	-	3622	43.52%	62.5	4.23	356	-

**Figure 2:**
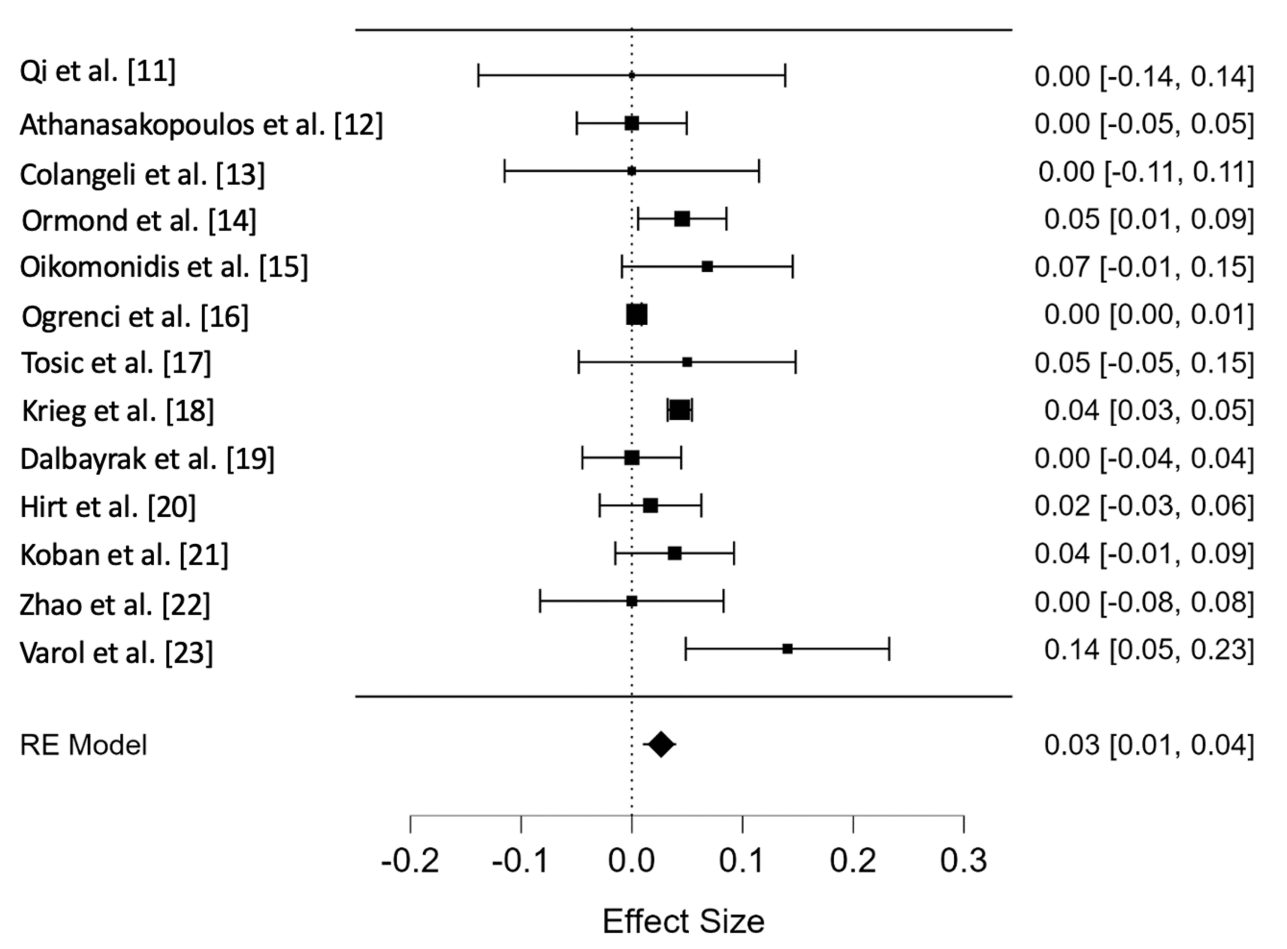
Forest plot showing effect size and 95% confidence interval for each study included in the PEEK rod group, as well as the overall effect size estimate. PEEK: polyetheretherketone; RE: random effects

**Figure 3:**
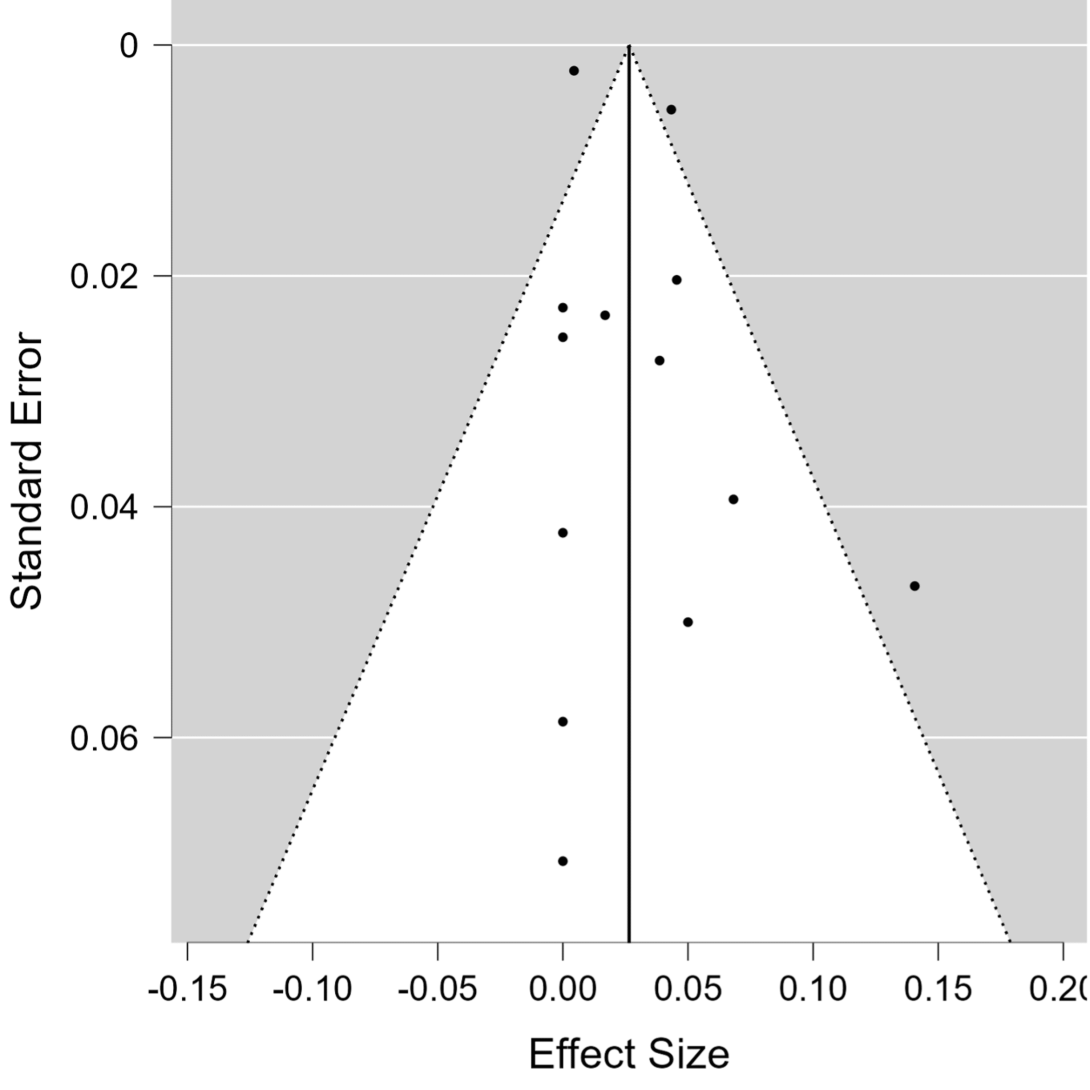
Funnel plot for the PEEK rod group. PEEK: polyetheretherketone

Meta-analysis of the PEEK rod group showed an overall incidence rate of ASD of 0.026 cases per person-year (95% CI = 0.010-0.043). The Forest plot representing the incidence rate of ASD, expressed in person-years, in each study, as well as the overall meta-analysis group, can be found in Figure 2. The PEEK rod group had moderate heterogeneity (I^2^ = 67.84%). The funnel plot for this group can be found in Figure 3, and the rank test for funnel plot asymmetry showed no evidence of publication bias (p = 0.858).

**Figure 4:**
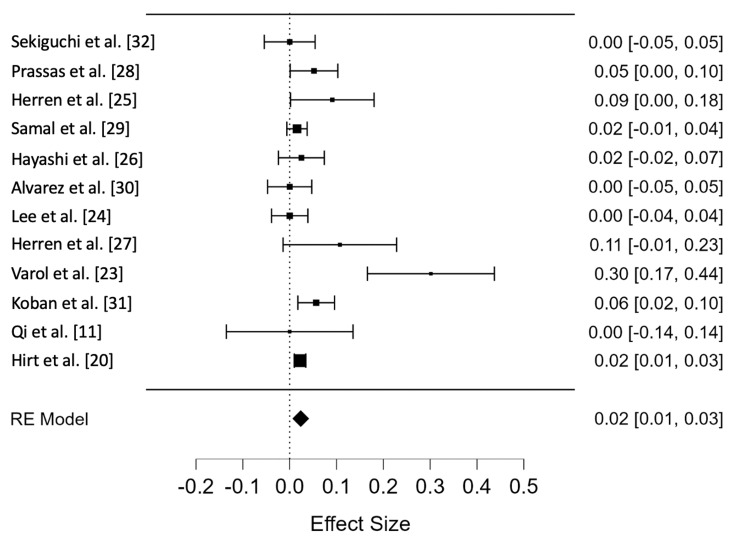
Forest plot for the titanium rod group. RE: random effects

**Figure 5:**
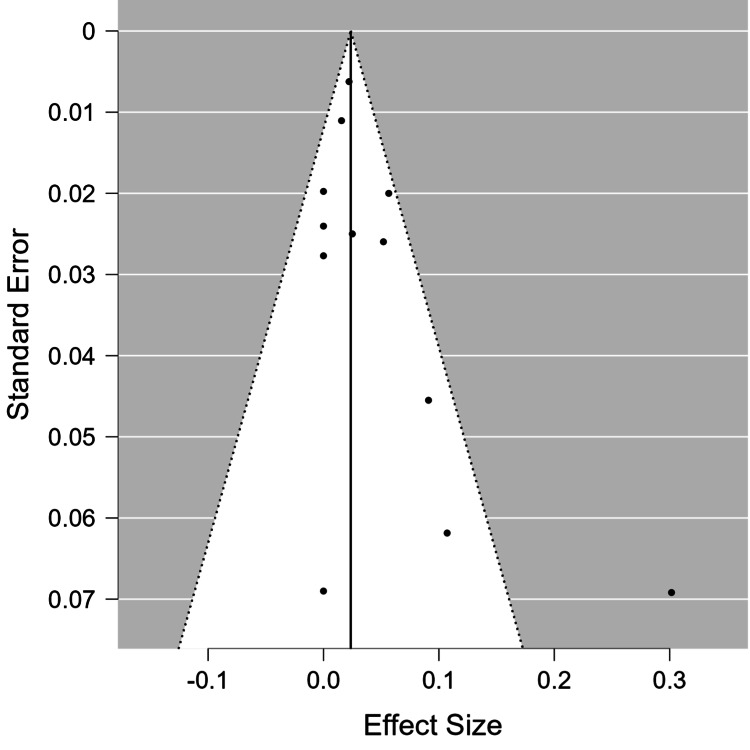
Funnel plot for the titanium rod group.

The random effects model showed that the overall incidence rate of ASD in the titanium rod group was 0.024 cases per person-year (95% CI = 0.014-0.033). Figure 4 shows the Forest plot for this group, revealing the overall incidence rate of ASD in this group as well as those of each study. The titanium rod group showed low heterogeneity (I^2^ = 0.36%), and the rank test for funnel plot asymmetry showed no evidence of publication bias (p = 0.116). Figure 5 shows the funnel plot for the titanium rod group.

**Figure 6:**
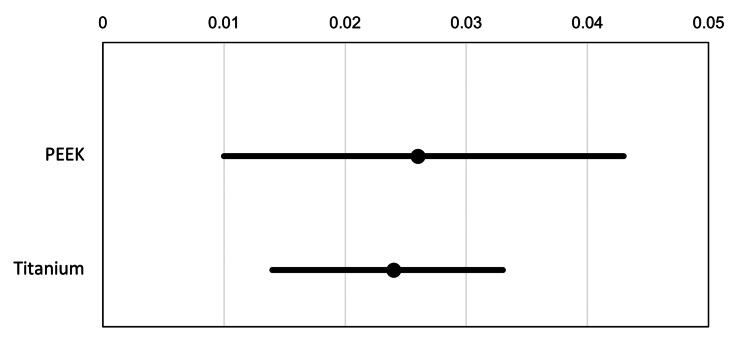
Comparison of overall ASD incidence rate estimates and 95% CIs. ASD: adjacent segment disease; CI: confidence interval; PEEK: polyetheretherketone

One-way ANOVA was performed to determine the significance of the difference between the two groups. The 95% CIs for each of the two groups are represented in Figure 6. The overall difference in the rate of ASD between the PEEK and titanium rod groups was 0.362. The ANOVA showed that the difference between the two groups was not statistically significant at the 5% level (p = 0.8422).

Discussion

The rate of posterior lumbar fusion has been rising. Between 2004 and 2015, the volume of lumbar fusions increased by 62.3% in the United States. Furthermore, this increase was found to be more pronounced in patients aged 65 and older, at 138.7%, and the proportion of procedures for which the indication was spondylolisthesis increased by 111%. Together, these two factors may be suggestive of an aging population, a trend that is predicted to continue over the coming years [32]. Because an older patient population is more likely to develop ASD, the development of spinal fusion systems that are associated with lower rates of ASD is critical to prevent rises in reoperation rates [3].

Since the 1990s, a new material has been used by some surgeons during spinal instrumentation, i.e., PEEK. It has multiple properties that make it attractive as a material for implants, such as radiolucency for improved postoperative imaging and a Young’s modulus of 3.6 GPa, which is much closer to that of cortical bone (12 GPa) than titanium (110 GPa) [33]. PEEK rods were introduced as part of a semi-rigid fixation system in 2007, and follow-up studies have shown that the Young’s modulus that is similar to cortical bone allows for less stress than with titanium rods [34,35]. One purported benefit of the use of PEEK over titanium rods is that this stress reduction may reduce the rate of ASD [36].

This is the first meta-analysis comparing the rates of ASD following posterior lumbar fusion with PEEK versus titanium rods. In total, 13 studies using PEEK rods and 12 studies using titanium rods were included in this analysis. The cohorts had similar sex distributions, though there was a significant difference in mean age between the two groups. The results of this meta-analysis showed that there was no significant difference between the rates of ASD between the PEEK rod and the titanium rod groups (p = 0.8422). This suggests that the material properties of PEEK may not be protective against ASD as predicted in some finite element studies and early clinical studies.

One of the major differences between the two cohorts was the mean follow-up time. Though not explicitly identified as a risk factor, it is plausible that the more time elapsed after fusion, the higher the annual incidence of ASD. Another major difference between the two groups was heterogeneity. The PEEK rod group had moderate heterogeneity (I^2^ = 67.84%), while the titanium rod group had low heterogeneity (I^2^ = 0.36%). This did not affect the estimates of the overall incidence rates of ASD within each group; however, this is a potential explanation for the much wider 95% CI calculated for the PEEK rod group [37]. This heterogeneity could be explained by greater inter-study variation in the presence of risk factors within the PEEK rod group.

Limitations

This study has some limitations. First, statistical analysis found that the PEEK group was statistically significantly younger than the titanium group (61.1 years vs. 62.5 years, p < 0.0001). Because age is a known risk factor for ASD, it is possible that this had some effect on the results of this study [3]. A second limitation of this study is the variability of the follow-up length within each group, as well as within each study. Although loss of patients to follow-up is expected, especially in prospective studies, this can potentially lead to changes in the calculated incidence rates. This can also affect raw case numbers, though this was somewhat corrected for by analyzing incidence rate in cases per person-year. However, there is some inherent error in this method, as some studies only reported a minimum, rather than a mean, follow-up time. Finally, a third limitation is the inconsistency of the definition of ASD, though most studies included here defined it as symptomatic ASD, there are some unclear definitions that could lead to the inconsistent inclusion of patients with radiographic ASD.

Further investigations comparing the incidence rates of ASD following fusion with PEEK rods as opposed to titanium rods should be large, prospective cohort studies utilizing matching to control for the presence of risk factors such as age, sex, osteoporosis, and preexisting injury to the facet joints. Furthermore, studies with longer follow-up periods could elucidate the long-term incidence of ASD following posterior lumbar fusion with PEEK rods. Finally, future meta-analyses on the topic could focus on comparing long-term overall complication and revision rates, including ASD, between patients fused with titanium rods and PEEK rods, as well as with other rod materials and dynamic stabilization systems.

## Conclusions

This is the first meta-analysis to compare ASD incidence following lumbar fusion with PEEK versus titanium rods. No statistically significant difference in ASD incidence was observed between the two materials. While PEEK offers biomechanical and imaging advantages, these do not appear to translate into lower clinical rates of ASD in the current literature. The findings emphasize the need for further high-quality studies that control for important clinical confounders such as age, osteoporosis, and preexisting facet joint disease. With a growing volume of lumbar fusions performed globally, optimizing hardware choice for long-term outcomes remains a priority in spinal surgery. The comparative efficacy of newer semi-rigid systems, dynamic stabilization constructs, and advanced material technologies deserves ongoing investigation.

## References

[1] Park P, Garton HJ, Gala VC, Hoff JT, McGillicuddy JE (2004). Adjacent segment disease after lumbar or lumbosacral fusion: review of the literature. Spine (Phila Pa 1976).

[2] Virk SS, Niedermeier S, Yu E, Khan SN (2014). Adjacent segment disease. Orthopedics.

[3] Kim DW, Kim JH, Ryu DS, Yoon SH (2020). The risk factors of early and late-onset adjacent segment degeneration after thoracolumbar fusion surgery. Nerve.

[4] Saavedra-Pozo FM, Deusdara RA, Benzel EC (2014). Adjacent segment disease perspective and review of the literature. Ochsner J.

[5] Seaman S, Kerezoudis P, Bydon M, Torner JC, Hitchon PW (2017). Titanium vs. polyetheretherketone (PEEK) interbody fusion: meta-analysis and review of the literature. J Clin Neurosci.

[6] Jin YJ, Kim YE, Seo JH, Choi HW, Jahng TA (2013). Effects of rod stiffness and fusion mass on the adjacent segments after floating mono-segmental fusion: a study using finite element analysis. Eur Spine J.

[7] Page MJ, McKenzie JE, Bossuyt PM (2021). The PRISMA 2020 statement: an updated guideline for reporting systematic reviews. BMJ.

[8] Walter SD, Yao X (2007). Effect sizes can be calculated for studies reporting ranges for outcome variables in systematic reviews. J Clin Epidemiol.

[9] Hanley JA, Lippman-Hand A (1983). If nothing goes wrong, is everything all right? Interpreting zero numerators. JAMA.

[10] Love J, Selker R, Marsman M (2019). JASP: graphical statistical software for common statistical designs. J Stat Softw.

[11] Qi L, Li M, Zhang S, Xue J, Si H (2013). Comparative effectiveness of PEEK rods versus titanium alloy rods in lumbar fusion: a preliminary report. Acta Neurochir (Wien).

[12] Athanasakopoulos M, Mavrogenis AF, Triantafyllopoulos G, Koufos S, Pneumaticos SG (2013). Posterior spinal fusion using pedicle screws. Orthopedics.

[13] Colangeli S, Barbanti Brodàno G, Gasbarrini A, Bandiera S, Mesfin A, Griffoni C, Boriani S (2015). Polyetheretherketone (PEEK) rods: short-term results in lumbar spine degenerative disease. J Neurosurg Sci.

[14] Ormond DR, Albert L Jr, Das K (2016). Polyetheretherketone (PEEK) rods in lumbar spine degenerative disease: a case series. Clin Spine Surg.

[15] Oikonomidis S, Ashqar G, Kaulhausen T, Herren C, Siewe J, Sobottke R (2018). Clinical experiences with a PEEK-based dynamic instrumentation device in lumbar spinal surgery: 2 years and no more. J Orthop Surg Res.

[16] Ogrenci A, Koban O, Yaman O, Yilmaz M, Dalbayrak S (2019). Polyetheretherketone rods in lumbar spine degenerative disease: mid-term results in a patient series involving radiological and clinical assessment. Turkish Neurosurg.

[17] Tosic L, Baschera D, Oberle J, Alex A (2019). Decompression and dynamic transpedicular stabilization using polyetheretherketone rods and pedicle screws vs. decompression alone for single-level spinal canal stenosis with listhesis: a retrospective case-control study. J Neurol Surg A Cent Eur Neurosurg.

[18] Krieg SM, Balser N, Pape H, Sollmann N, Albers L, Meyer B (2020). Topping-off technique for stabilization of lumbar degenerative instabilities in 322 patients. J Neurosurg Spine.

[19] Dalbayrak S, Öğrenci A, Akar E, Koban O, Yılmaz A, Yılmaz M (2020). Clinical and radiological outcomes after correction of degenerative lumbar scoliosis with dynamic stabilization (with the help of a rigid rod); and describing an alternative technique. J Clin Neurosci.

[20] Hirt D, Prentice HA, Harris JE (2021). Do PEEK rods for posterior instrumented fusion in the lumbar spine reduce the risk of adjacent segment disease?. Int J Spine Surg.

[21] Koban O, Öğrenci A, Akar EA, Uyanık AS, Yılmaz M, Dalbayrak S (2021). Radiological and clinical comparisons of the patients with rheumatoid arthritis operated with rigid and dynamic instrumentation systems due to lumbar degenerative spinal diseases. J Orthop Sci.

[22] Zhao Y, Xu B, Qi L (2022). Hybrid surgery with PEEK rods for lumbar degenerative diseases: a 2-year follow-up study. BMC Musculoskelet Disord.

[23] Varol E, Etli MU, Avci F, Yaltirik CK, Ramazanoglu AF, Onen MR, Naderi S (2022). Comparison of clinical and radiological results of dynamic and rigid instrumentation in degenerative lumbar spinal stenosis. J Craniovertebr Junction Spine.

[24] Lee SM, Lee GW (2015). The impact of generalized joint laxity on the clinical and radiological outcomes of single-level posterior lumbar interbody fusion. Spine J.

[25] Herren C, Simons RM, Bredow J (2018). Posterior lumbar interbody fusion versus dynamic hybrid instrumentation: a prospective randomized clinical trial. World Neurosurg.

[26] Hayashi K, Toyoda H, Terai H (2018). Comparison of minimally invasive decompression and combined minimally invasive decompression and fusion in patients with degenerative spondylolisthesis with instability. J Clin Neurosci.

[27] Herren C, Sobottke R, Pishnamaz M (2018). The use of the DTO™ hybrid dynamic device: a clinical outcome- and radiological-based prospective clinical trial. BMC Musculoskelet Disord.

[28] Prassas A, Alexiou GA, Pourni P, Magras J, Tsoleka K, Tsonidis CA, Tsitsopoulos PP (2021). Clinical outcome following decompression and short or long instrumented fusion in lumbar degenerative spinal stenosis. A prospective case-control analysis. Clin Neurol Neurosurg.

[29] Samal F, Sterba A, Haninec P, Jurek P, Waldauf P, Filip M, Linzer P (2021). Long-term outcome after midline lumbar fusion for the treatment of lumbar spine instability due to degenerative disease. World Neurosurg.

[30] Alvarez R, Chinea A, Braley A (2021). Cortical screw fixation using CT-navigation coupled with real-time electrophysiological monitoring of individual screw placement for unstable degenerative lumbar spondylolisthesis. Interdisc Neurosurg.

[31] Sekiguchi I, Takeda N, Ishida N (2022). Indirect decompression of the central lumbar spinal canal by means of simultaneous bilateral transforaminal lumbar interbody fusion for severe degenerative lumbar canal stenosis with 3 years minimum follow-up. Interdisc Neurosurg.

[32] Martin BI, Mirza SK, Spina N, Spiker WR, Lawrence B, Brodke DS (2019). Trends in lumbar fusion procedure rates and associated hospital costs for degenerative spinal diseases in the United States, 2004 to 2015. Spine (Phila Pa 1976).

[33] Vadapalli S, Sairyo K, Goel VK, Robon M, Biyani A, Khandha A, Ebraheim NA (2006). Biomechanical rationale for using polyetheretherketone (PEEK) spacers for lumbar interbody fusion-a finite element study. Spine (Phila Pa 1976).

[34] Highsmith JM, Tumialán LM, Rodts GE Jr (2007). Flexible rods and the case for dynamic stabilization. Neurosurg Focus.

[35] Chou WK, Chien A, Wang JL (2015). Biomechanical analysis between PEEK and titanium screw-rods spinal construct subjected to fatigue loading. J Spinal Disord Tech.

[36] Selim A, Mercer S, Tang F (2018). Polyetheretherketone (PEEK) rods for lumbar fusion: a systematic review and meta-analysis. Int J Spine Surg.

[37] Imrey PB (2020). Limitations of meta-analyses of studies with high heterogeneity. JAMA Netw Open.

